# Complete and Assembled Genome Sequence of *Staphylococcus aureus* RKI4, a Food-Poisoning Strain Exhibiting a Novel *S. aureus* Pathogenicity Island Carrying *seb*

**DOI:** 10.1128/genomeA.00769-15

**Published:** 2015-07-02

**Authors:** Marc J. A. Stevens, Roger Stephan, Sophia Johler

**Affiliations:** a Laboratory of Food Biotechnology, Institute of Food, Nutrition, and Health, ETH Zurich, Zurich, Switzerland; b Vetsuisse Faculty, Institute for Food Safety and Hygiene, University of Zurich, Zurich, Switzerland

## Abstract

The genome of *Staphylococcus aureus* RKI4, a strain isolated from feces of a patient in a case of staphylococcal food poisoning, was sequenced using combined Illumina and single-molecule real-time sequencing. Hierarchical assembly of the genome resulted in a 2,725,654-bp chromosome and a 17,905-bp mobile genetic element.

## GENOME ANNOUNCEMENT

*Staphylococcus aureus* can cause staphylococcal food poisoning, with an estimated 240,000 cases occurring each year in the United States alone (1). Upon oral intake of staphylococcal enterotoxins, patients show signs of acute gastroenteritis, including violent vomiting and diarrhea. In this study, we present the complete genome sequence of *S. aureus* RKI4. The strain was isolated from feces of a 37-year-old patient suffering from staphylococcal food poisoning in Germany in 2008. The strain exhibits a novel *S. aureus* pathogenicity island (SaPI) carrying the *seb* gene encoding staphylococcal enterotoxin B. SaPIs are phage-related chromosomal islands and represent *S. aureus* mobile genetic elements. The novel SaPI described in this study exhibits the att site core 5′ ATT TTA CAT CAT TCC TGG CAT 3′. Production of enterotoxin B was confirmed using the SET-RPLA kit (Oxoid, Basel, Switzerland), and the strain was assigned to clonal complex 9 and *spa* type t733(2).

The genome of RKI4 was sequenced using a combination of Illumina HiSeq 2000 and PacBio single-molecule real-time sequencing (SMRT) technologies. The SMRT sequencing resulted in 104,146 reads with a mean length of 3,447 bp. The reads were assembled using the Hierarchical Genome Assembly Process (HGAP) (3). Duplicate sequences at the end of contigs were combined and the assembly resulted in two contigs of 2,725,653 and 17,905 bp, both with an average coverage of 102-fold. Illumina HiSeq 2000 sequencing resulted in 90,949,143 reads of 50-bp length. These reads were mapped to the 2 contigs obtained via HGAP using the CLC Genomics Workbench version 8.0 (CLC Bio, Aarhus, Denmark). A total of 88,702,834 reads could be mapped to contig 1, corresponding to a coverage of 1,627-fold. In addition, 2,390,850 reads were mapped to contig 2, resulting in a coverage of 6,627-fold. The Illumina reads were used to correct the SMRT reads, resulting in the complete assembled genome of *S. aureus* RKI4. The genome consists of a 2,725,654-bp chromosome and a 17,905-bp mobile genetic element with GC contents of 32.83 and 28.06%, respectively. Annotation was performed using the NCBI Prokaryotic Genomes Automatic Annotation Pipeline (4). RKI4 contains 2,631 predicted open reading frames, including 59 tRNA genes and 6 rRNA operons. The complete genome of RKI4 will contribute to further understanding of virulence and genome plasticity within *S. aureus*.

### Nucleotide sequence accession numbers. 

Sequence and annotation data of the complete genome of *S. aureus* strain RKI4 were deposited in the GenBank database with the accession numbers CP011528 for the chromosome and CP011529 for the mobile genetic element.
